# Alginate-Sr/Mg Containing Bioactive Glass Scaffolds: The Characterization of a New 3D Composite for Bone Tissue Engineering

**DOI:** 10.3390/jfb15070183

**Published:** 2024-07-02

**Authors:** Benedetta Guagnini, Barbara Medagli, Bianca Zumbo, Valeria Cannillo, Gianluca Turco, Davide Porrelli, Devis Bellucci

**Affiliations:** 1 Department of Medicine, Surgery and Health Sciences, University of Trieste, Piazza dell’Ospitale 1, 34129 Trieste, Italy; benedetta.guagnini@phd.units.it (B.G.); bmedagli@units.it (B.M.); bianca.zumbo@phd.units.it (B.Z.); gturco@units.it (G.T.); 2 Department of Engineering “Enzo Ferrari”, University of Modena and Reggio Emilia, Via P. Vivarelli 10, 41125 Modena, Italy; valeria.cannillo@unimore.it (V.C.); devis.bellucci@unimore.it (D.B.); 3 Department of Life Sciences, University of Trieste, Via Alexander Fleming 31/B, 34127 Trieste, Italy

**Keywords:** alginate, bioactive glass, bioactivity, bone regeneration, porous scaffolds, magnesium, strontium

## Abstract

In bone regeneration, combining natural polymer-based scaffolds with Bioactive Glasses (BGs) is an attractive strategy to improve the mechanical properties of the structure, as well as its bioactivity and regenerative potential. Methods: For this purpose, a well-studied alginate/hydroxyapatite (Alg/HAp) porous scaffold was enhanced with an experimental bioglass (BGMS10), characterized by a high crystallization temperature and containing therapeutic ions such as strontium and magnesium. This resulted in an improved biological response compared to 45S5 Bioglass^®^, the “gold” standard among BGs. Porous composite scaffolds were fabricated by freeze-drying technique and characterized by scanning electron microscopy and microanalysis, infrared spectroscopy, and microcomputed tomography. The mechanical properties and cytocompatibility of the new scaffold composition were also evaluated. The addition of bioglass to the Alg/HAp network resulted in a slightly lower porosity. However, despite the change in pore size, the MG-63 cells were able to better adhere and proliferate when cultured for one week on a BG scaffold compared to the control Alg/HAp scaffolds. Thus, our findings indicate that the combination of bioactive glass BGMS10 does not affect the structural and physicochemical properties of the Alg/HAp scaffold and confers bioactive properties to the structures, making the Alg/HAp-BGMS10 scaffold a promising candidate for future application in bone tissue regeneration.

## 1. Introduction

Bone defects and injuries severely impact healthcare systems. Despite good bone regeneration capacity, in the case of extensive damage related to trauma or disease, advanced age, or other concomitant pathologies, the regeneration process is unable to restore complete tissue integrity. Current approaches to replacing or restoring bone tissue come with several potentially harmful drawbacks [1]. Bone Tissue Engineering (BTE) is a promising strategy to repair and regenerate damaged tissues by combining stem cells, innovative scaffolds, and biological factors to achieve complete bone regeneration and the restoration of bone functions.

Most common bone substitutes consist of synthetic and natural materials that mimic the physiological environment of bone, creating a temporary artificial microenvironment with excellent advantages in terms of biocompatibility, osteoinduction, and osteoconduction [2]. Owing to their ability to form hydrogels and porous structures, alginate-based scaffolds have emerged in tissue engineering applications due to their biocompatibility, low toxicity, low cost, and easy manufacturing [3,4,5].

Alginate, a natural polysaccharide derived from marine algae (Laminaria Hyperborea, Laminaria Digitatam, and Ascophyllum Nodosum) consists of an alternating chain of β-D-mannuroinc acid (M residues) and α-D-guluronic acid (G residues). The alginate chains, interacting with bivalent cations (e.g., Ca^2+^, Sr^2+^, and Ba^2+^), are able to form hydrogels thanks to a cooperative process in which the G monomers, due to their affinity for calcium ions, provide a stable site for the formation of crosslinking between two polymer chains, forming so-called egg boxes [5]; the freeze drying of the hydrogels allows interconnected porous structures to be obtained [6]. These porous structures are particularly suitable for bone tissue engineering applications, as they favor the proliferation and spread of cells at the depth of the scaffold. The ideal porosity level for the scaffold is around 90% of its volume. This range not only maximizes the surface area available for cell colonization, attachment, and proliferation but also enables the exchange of metabolites and catabolites [7].

Despite these promising properties, alginate scaffolds exhibit poor mechanical properties, bioactivity, and osteoconductivity and are not able to promote bio-mineralization, which is an important feature for bone regeneration. Thus, these materials have been combined with ceramic fillers in attempts to improve the mechanical and biological properties of the structures [8] and to improve interactions with surrounding tissues.

Through the years, composite scaffolds based on alginate (Alg) have been implemented with hydroxyapatite (HAp), the inorganic component of bone tissue, and have been optimized to obtain a porous structure that promotes osteoconductivity and improves strength to high deformation, in addition to supporting attachment and proliferation of osteoblasts [9]; these scaffolds have been further modified to promote their osteoconductivity by adding cell-adhesive polymers [10] or to confer antimicrobial activity by adding silver nanoparticles [11]. Therefore, Alg/HAp scaffolds represent a simple but well-characterized scaffold model to analyze the effects of bioactive elements introduced into these scaffolds.

Nowadays, attention is focused on bioactive glasses (BGs), which are investigated for their bioactive potential in bone formation. When combined with natural polymers, BGs enhance the system’s bioactivity thanks to the presence of active ions, whose concentrations can be adjusted to trigger specific molecular responses in the host, favoring regenerative processes [12,13]. The dissolution products of BGs can promote gene expression in osteoblast cells and angiogenesis, which makes them highly effective in forming a strong bond between the scaffolds and the hard and soft tissues [14].

Although 45S5 Bioglass^®^ (45S5^®^) (composition, in mol%: 24.4 Na_2_O; 26.9 CaO; 2.6 P_2_O_5_; 46.1 SiO_2_) remains the “gold-standard” choice in the fields of dentistry and bone tissue engineering due to its remarkable capacity to create natural bonds with mineralized bone tissue [15], the BG prepared by Hench and his team in the 1970s does face certain limitations. For instance, it tends to crystallize when subjected to the thermal treatments essential for creating sintered materials such as porous scaffolds, potentially decreasing the biological efficacy of the end product [16,17]. Furthermore, over time, an ever-increasing number of investigations has emerged in the literature, emphasizing the significant biological effects of various metallic ions, such as strontium, magnesium, zinc, copper, or silver [18,19]. Depending on the type of ion, the effect ranges from stimulating angiogenesis and osteogenesis to promoting the adhesion of specific proteins to the biomaterial, even conferring antibacterial properties to the biomaterial. These ions are not present in the composition of 45S5^®^. Therefore, in recent years, research has invested significant efforts in producing new BGs containing biologically active metallic ions tailored for specific applications.

A previously developed bioactive glass, BGSM10 (composition, in mol%: 2.3 Na_2_O; 2.3 K_2_O; 25.6 CaO; 10.0 MgO; 10.0 SrO; 2.6 P_2_O_5_; 47.2 SiO_2_), shows particular promise due to its lower tendency to crystallize, its relatively low sintering temperature with respect to 45S5^®^, and its high bioactivity [20]. BGSM10 incorporates biologically active ions such as magnesium and strontium to optimize its therapeutic potential. Magnesium plays an important role in bone metabolism, promoting stem cell growth and differentiation and enhancing the mechanical behavior of newly formed bone [21,22]. Strontium has been demonstrated to enhance osteogenesis and osteoblast differentiation, contributing significantly to bone turnover in a way that favors bone formation. This leads to increased bone mass and strength [23]. The excellent sinterability of BGMS10, combined with the biological potential of the ions it contains, has allowed for the successful utilization of this promising BG in various types of composite materials [24,25].

Recent literature has shown that by incorporating BG containing Sr ions into an alginate structure, the mechanical performances of alginate/BG scaffolds can be increased compared to structures made solely of alginate [26]. Furthermore, the release of Mg^2+^ and SiO^4−^ ions from bioglass has a synergistic effect that promotes bone turnover by increasing the adhesion, proliferation, and differentiation of osteoblast cells [27]. This leads to an increase in the level of alkaline phosphatase and other biomarkers of differentiation and cell cycle regulation [28,29]. Despite the advantages offered by composite scaffolds used as bone grafts, surgical infections remain a critical issue for patients who undergo bone implant procedures. The lack of antibacterial activity in composite scaffolds is still a drawback, which is a significant area of concern. In recent years, many BGs containing therapeutic ions known for their antibacterial properties, such as copper [30], zinc [31], silver [32], and strontium (which also exhibits mild antibacterial activity) [33], have emerged.

This study aimed to synthesize a novel, biocompatible composite material with sufficient porosity and features suitable for bone scaffold applications employing a simple, reproducible freeze-drying technique. Specifically, we present a composite material made of alginate and hydroxyapatite combined with BGMS10. The primary goal of the study is to determine whether the addition of BGMS10 affects the structure and physicochemical properties of the alginate–hydroxyapatite scaffold. In addition, a preliminary characterization was conducted using 45S5^®^ to investigate the effect of the commercial glass on the alginate structure. As previously mentioned, incorporating a glass filler serves to enhance the strength of a pure alginate scaffold and stimulate bioactivity, potentially facilitating in situ osseointegration [23]. The synergic effects of Mg and Sr ions contained in BGMS10 were evaluated in terms of the mechanical performance and biocompatibility of the composite scaffold. The viability and proliferation of the osteosarcoma cell line (MG-63) on the composite scaffold were compared to Alg/HAp scaffolds used as a reference. Furthermore, the novel combination of BGMS10 glass and alginate to achieve synergistic effects of Sr^2+^ and Mg^2+^ with Ca^2+^ can be investigated to prevent bacterial colonization of the scaffolds after implantation. In this regard, the potential antimicrobial activity provided by the addition of BGMS10 was investigated with respect to *Staphylococcus aureus* and *Escherichia coli*.

## 2. Materials and Methods

### 2.1. Materials

Sodium alginate (Alg, FG = 0,67; FGG = 0,59; MW = 135,000) derived from Laminaria Hyperborea was provided by FMC Biopolymers (Drammen, Norway). Raw materials for bioactive glass (BG) preparation were purchased from Carlo Erba (Milan, Italy). Simulated Body Fluid (SBF) salts (NaCl, NaHCO_3_, KCl, K_2_HPO_4_, MgCl_2_, CaCl_2_, and Na_2_SO_4_), phosphate-buffered saline (PBS), hydroxyapatite micrometric powder (HAp), Luria–Bertani broth (LB), glucono-delta-lactone (GDL), and the In Vitro Toxicology Assay Kit, Resazurin based Tox8 and Tetrazolium salts test (MTT) were purchased from Merck (St. Louis, MI, USA). Dulbecco’s Modified Eagle Medium (DMEM), fetal bovine serum, L-glutamine, and penicillin/streptomycin were purchased from EuroClone (Milan, Italy).

### 2.2. Synthesis of Bioactive Glasses and Chemical Characterization of Bioactive Glasses and HAp

The bioactive glasses investigated in this study, specifically 45S5^®^ and BGMS10, were produced using a conventional melt-quenching method as described in previous literature [20]. In brief, raw powdered materials were melted in a platinum crucible at 1450 °C. The thermal cycle involved heating from room temperature to 1100 °C at a rate of 10 °C/min, with a one-hour hold at 1100 °C to aid in the decomposition of raw carbonate materials. Subsequently, the temperature was increased at a rate of 10 °C/min until reaching 1450 °C. The molten glass was then quenched in room-temperature water to form a frit, which was dried in an oven at 110 °C for 12 h. Following drying, the 45S5^®^ and BGMS10 frits underwent milling for 45 min in jars with alumina balls and were sieved to achieve the final particle size (<63 microns).

### 2.3. Preparation of Alginate/Hydroxyapatite-Bioactive Glass Composite Scaffolds

Alg (2% *w*/*v*)/HAp (3% *w*/*v*) scaffolds were prepared as previously reported by Turco et al. 2009 [9]. Alginate was solubilized overnight at room temperature in a deionized water volume corresponding to 70% of the final volume of the gel. Then, a suspension of the micrometric powder of HAp was prepared by stirring for 30 min in a deionized water volume corresponding to 20% of the final volume of the gel. The HAp suspension was then combined with the Alg solution and stirred for approximately 30 min. Subsequently, GDL (60 mM) was solubilized in a deionized water volume corresponding to 10% of the final volume of the gel and immediately poured into the Alg/HAp mixture. After 60 s of stirring, the mixture was poured into the wells of a 24-well plate and left overnight sealed at room temperature for alginate gelation. The day after, the hydrogels were frozen from room temperature to −20 °C, lowering the temperature by 1 °C every 4 min using a cryostat (circulating bath 28L, VWR, Radnor, PA, USA). Then, the hydrogels were freeze-dried (ALPHA 1–2 LD plus freeze dryer, CHRIST, Osterode am Harz, Germany) for 48 h.

Starting from the previously described procedure, the inorganic portion represented by hydroxyapatite was partially substituted with different concentrations of BGs, namely BGMS10 or 45S5^®^. An adequate amount of the BG was weighted to reach the final concentration of 0.3% (*w*/*v*) or 0.6% (*w*/*v*) of the total gel volume, corresponding to 10% or 20% of the inorganic mineral part, respectively. The BGs were added in water suspensions of HAp at concentrations of 2.7% or 2.4% *w*/*v* of the total gel volume, respectively. At the end of the preparation, the overall composition of dried scaffolds consisted of 40% *w*/*w* alginate, 48 or 54% *w*/*w* HAp and 6 or 12% *w*/*w* of the BG. 

To replicate and study the effect of sodium ions on the alginate/HAp/45S5^®^ scaffold’s polymerization, NaCl was added to the HAp-BGSM10 solution. The obtained dried scaffolds, whose composition is reported in Table 1, were named as follows: Alginate/HAp-BGMS10 6%, BG6-sc; Alginate/HAp-BGMS10 12%, BG12-sc; Alginate/HAp-45S5^®^ 6%, 45S5^®^6-sc; Alginate/HAp scaffolds, Ctrl-sc; Alginate/HAp-BGMS10 (adding to the solution for the hydrogel, NaCl 10% *w*/*v*), BG6d-sc.

### 2.4. PhysicoChemical and Structural Scaffold Characterizations

#### 2.4.1. X-ray Microcomputed Tomography Analysis

X-ray microcomputed tomography of the scaffolds was performed using the TOMOLAB cone beam system (Elettra-Sincrotrone, Trieste, Italy). Samples were placed onto the rotating stage of the instrument, and acquisitions of the projections were performed using the following parameters: source–detector distance (FDD), 25 cm; source–sample distance (FOD), 8 cm; magnification, 3.1×; binning, 2 × 2; resolution, 8 μm; tomography dimensions (pixels), 2004 × 1335; slice dimensions (pixels), 1984 × 1984; number of tomographies, 1440; number of slices, 1332; E = 40 kV; I = 200 μA; exposure time, 1.5 s. Nrecon commercial software (version 1.7) was used for the slice reconstruction process and for beam hardening and ring artifact corrections. Slice segmentation was performed according to Otsu’s method [34] using Fiji software (Version 2.13.0) [35]. The BoneJ plugin (release 7.0.15) [36] was then used to analyze the morphological parameters (porosity, trabecular thickness, and trabecular spacing). The quantification of morphological parameters was performed on cubic Volumes Of Interest (VOIs) with 3.5 mm sides.

#### 2.4.2. Scanning Electron Microscopy (SEM)

Scaffold morphology was examined by SEM investigations. Samples were sectioned with a razor blade on various planes to visualize the cross and top sections. Then, they were mounted on aluminum stubs covered with a double-sided carbon tape and visualized by a scanning electron microscope (Quanta250 SEM, FEI, Hillsboro, OR, USA) operating in environmental mode. The working distance was adjusted to obtain the suitable magnification; the acceleration voltage was set to 30 kV.

#### 2.4.3. Attenuated Total Reflectance–Fourier Transform Infrared (ATR-FTIR) Spectroscopy

ATR-FTIR was performed to analyze the presence of BGs and HAp within the scaffolds. IR spectra were acquired with a Nicolet iS50 spectrometer (Thermo Scientific, Milan, Italy) within a 525–4000 cm^−1^ wavenumber range. Three samples were analyzed for each condition, acquiring a spectrum with 32 scans and a resolution of 0.482 cm^−1^. Three independent scaffolds were analyzed for each condition.

#### 2.4.4. Ion Release Evaluation with Energy-Dispersive Spectroscopy (EDS)

Ion release was evaluated by EDS. Scaffolds were immersed in PBS in a 24-well plate and incubated at room temperature. After the selected time points (1 h, 8 h, and 24 h), samples were air-dried and placed on aluminum stubs covered with double-sided carbon tape and coated with a thin layer of carbon using the Q150T ES plus sputter coater (Quorum Technologies, Lewes, UK). Samples were then analyzed using a Gemini300 field-emission scanning electron microscope (Zeiss, Oberkochen, Germany), equipped with an XFlash 610M EDS probe (Bruker, Billerica, MA, USA) at an 8.5 mm working distance with an acceleration voltage of 10 kV.

### 2.5. Swelling Studies

Ctrl-sc, BG6-sc, and BG12-sc (N = 12) were weighed and immersed in 4 mL of PBS in a 12-well plate at 37 °C. At the selected time points (10 min, 30 min, 1 h, 2 h, 4 h, and 24 h), the scaffolds were gently removed from the PBS and placed on 8 layers of blotting paper for 10 s to remove excess PBS. Scaffolds were then weighed, and weight variation was calculated according to Equation (1):

Swelling (%) = ((Ws − Wd)/Wd) × 100 (1)

where Wd and Ws represent scaffold weights in the dry and the swollen condition, respectively. The results were taken as the average of four measurements. The scaffolds were then soaked again in the same wells containing PBS.

### 2.6. Uniaxial Compression Tests of Scaffolds

A universal testing machine (GaldabiniSun 500, Cardano al Campo, VA, Italy) coupled with a 100 N load cell was used to test Ctrl-sc, BG6-sc, and BG12-sc (n = 18). Dry scaffolds and scaffolds soaked in PBS for 10 min and 1 week were tested. A deformation rate of 1 mm/min was applied down to a 7 mm displacement. The compressive modulus was calculated in the 1–5% strain range (within the linear behavior of the material) of the stress–strain curves using a custom-made data analysis system developed in the laboratory.

### 2.7. Cell Culture, Adhesion, and Proliferation on Scaffolds

Biological tests were performed on human MG-63 osteoblast cells (ATCC number: CRL1427). Cells were cultured in high-glucose DMEM (Dulbecco’s Modified Eagle Medium) (EuroClone) supplemented with 2 mM l-glutamine, 10% *v*/*v* fetal bovine serum (FBS), 100 U/mL penicillin, and 0.1 mg/mL streptomycin and maintained in a 25 cm^2^ culture flask at 37 °C in a humidified 5% CO_2_ atmosphere.

For the 3D cell culturing, the scaffolds were cut at their extremities with a razor blade and sterilized by UV irradiation (30 min per 3 times). Once sterilized, they were soaked in 5 mM CaCl_2_, 100 U/mL penicillin, and 100 μg/mL streptomycin for 20 min (to stabilize the structure and remove residual reagents), then in a 24-well plate equilibrated for 24 h in complete high-glucose DMEM.

Before cell seeding, the medium was removed, and MG-63 cells (40,000 cells per scaffold resuspended in 40 μL of complete high-glucose DMEM) were gently seeded on the top of the scaffold (scaffold diameter, 10 mm). After 4 h of incubation at 37 °C in a humidified 5% CO_2_ atmosphere, 2 mL of culture medium was slowly added to each well, and the medium was replaced every 3 days. Cell adhesion was evaluated after 24 h by Alamar Blue, and the proliferation was monitored for 10 days using Alamar and MTT assay.

#### 2.7.1. Alamar Blue

The metabolic activity of cells seeded into the scaffolds was quantitatively measured with the Alamar Blue test. At each time point, all DMEM was removed, and scaffolds were transferred in a new 24-well culture plate (well diameter, 14.5 mm) to avoid also testing the cells adhered on the bottom of the wells. Ctrl-sc, BG-03 sc, and BG-06 sc were treated with Alamar Blue (Merck, St. Louis, MI, U.S.A.) diluted 1:30 in high-glucose DMEM (700 µL per scaffold). The plate was incubated in the dark for 4 h at 37 °C under 5% CO_2_. After incubation, 200 µL per sample of the supernatant was transferred to a black 96-well plate, and fluorescence intensity (excitation wavelength: 544 nm; emission wavelength: 590 nm) was measured by means of a FLUOStar^®^ Omega-BMG Labtech spectrophotometer. The fluorescence readings were normalized with the values obtained from an empty scaffold (blank). Scaffolds were washed with PBS to remove the Alamar Blue solution, and fresh, fully supplemented medium was added to each well.

#### 2.7.2. MTT Assay

A tetrazolium salt test (MTT) (Merck, St. Louis, MI, USA) was used to evaluate cell metabolic activity. At the selected time point, a solution of MTT diluted 1:5 in high-glucose DMEM (700 µL per scaffold) was added and incubated for 4 h at 37 °C under 5% CO_2_ in the dark. Purple formazan was eluted in 2 mL dimethyl sulfoxide (DMSO) for 30 min at room temperature. Samples were cut into four parts and squeezed using a tip to allow for better dissolution of Formazan’s crystal into the scaffold. Subsequently, the supernatant was collected in a test tube and centrifuged to eliminate scaffold residues that could interfere with the absorbance signal. At the end, 200 μL was transferred in a transparent 96-well plate, and absorbance (OD = 560 nm) was measured by a spectrophotometer. The absorbance of the formed formazan product was directly proportional to the living cells on the porous scaffold. Cell-free scaffolds were used as blanks. The entire experiment was incubated for 1, 3, 7, and 10 days after seeding to study the proliferation of the cells.

#### 2.7.3. Cell Morphology Study by Stereoscope and eSEM Observation

To evaluate cell colonization, samples were treated with 700 μL per scaffold MTT for 4 h (as described in Section 2.7.2), but the formazan crystals were not eluted. Insoluble crystals are a signal of a viable cluster of cells and can be detected by a stereoscope (Leica MZ16) at different magnifications. Image processing and analysis were performed by Image Pro 6.2 software.

The scaffolds were also analyzed by SEM using a Quanta 250 SEM (FEI) working under environmental conditions with an acceleration voltage of 30 kV to investigate MG-63 morphology on day 1 and day 8 after seeding.

### 2.8. Antimicrobial Effects of Composite Scaffolds

The scaffolds were sterilized by UV irradiation (30 min, repeated 3 times). Once sterilized, they were soaked in 5 mM CaCl_2_ for 10 min to stabilize the structure and washed in PBS to remove reagent residues. The sterile scaffolds were added to 4 mL of LB broth (50 mg/mL) and incubated for 24 h at 37 °C; the following day, the media were centrifuged to remove scaffold residuals and successively used to culture *Staphylococcus aureus* (ATCC 25923) and *Escherichia coli* (ATCC 25922) to test for possible antibacterial properties.

Bacteria were harvested from a glycerol pellet stored at −80 °C and resuspended in 5 mL of Luria–Bertani (LB) broth; the bacterial suspensions were incubated overnight at 37 °C under agitation (140 rpm). The following day, 300 µL of the overnight cultures was re-inoculated in 10 mL of fresh LB broth. Then, bacteria were incubated again at 37 °C and 140 rpm until an optical density of 0.3 600 nm (OD600) was reached (mid.log phase), and the bacterial concentration was evaluated via predictive models, knowing that OD600 = 0.29 corresponds to a bacterial concentration of 1.8 × 10^8^ CFU/mL for *S. aureus* ATCC 25923 and OD600 = 0.3 corresponds to a bacterial concentration of 1.15 × 10^8^ CFU/mL for *E. Coli* ATCC 25922.

In a 96-well plate, bacterial suspensions (105 CFU/mL) were grown in LB broth conditioned scaffold powder and analyzed at 600 nm every 30 min for 8 h at 37 °C using a microplate spectrophotometer (InfinitieM200PRO NanoQuant, Tecan, Männedorf, Switzerland). The results were obtained by averaging the values of replicates.

### 2.9. Statistical Analysis

Statistical analyses were performed using GraphPad software (version 8.0.2, Insight Partners, New York, NY, USA). The Shapiro–Wilk test was used to analyze the distribution of the data, which were analyzed by means of two-way analysis of variance (ANOVA), applying Bonferroni’s correction if the data followed a normal distribution. Data that were not assumed to be normally distributed were tested using Kruskal–Wallis and Mann–Whitney non-parametric tests, applying Bonferroni’s correction. Statistical significance was set at α = 0.05.

## 3. Results

### 3.1. Preparation and Characterization of Composite Scaffolds

**Figure 1:**
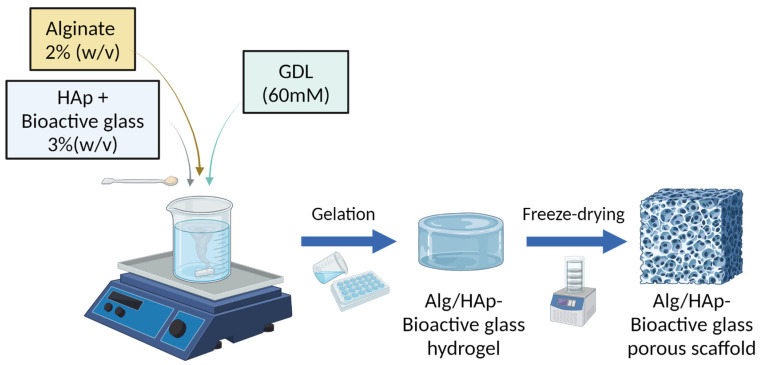
Representation of the composite scaffold preparation process. Alginate (Alg) hydrogel containing hydroxyapatite (HAp) and bioactive glass is prepared through a slow gelation method using glucono-delta-lactone (GDL); the hydrogel is then freeze-dried to obtain the porous scaffold. Created with biorender.com, accessed on 5 February 2024.

Three-dimensional porous scaffolds were obtained by freeze drying hydrogels of alginate/HAp (Ctrl-sc) and BGMS10-alginate/HAp prepared using a slow gelation method [9]. Two different concentrations of BGMS10 were used, namely 0.3% and 0.6% (*w*/*v*), lowering the concentration of HAp so as not to alter the total mineral content. Figure 1 shows the composite scaffold manufacturing process. The same procedure was performed with the 45S5 Bioglass ^®^ to compare the use of BGMS10 within the scaffold.

**Figure 2:**
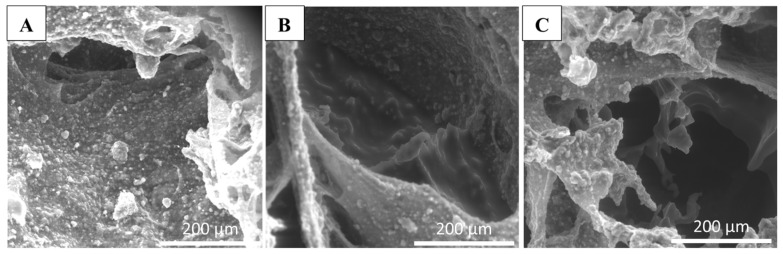
Morphological characterization of scaffolds. eSEM analysis of the structures of Ctrl-sc (**A**), BG6-sc (**B**), and BG12-sc (**C**) taken at 300× magnification. Differences in the surface roughness between samples with and without BGMS10 bioglass can be appreciated.

The morphological characterization of the scaffolds was performed using Environmental Scanning Electron Microscopy (eSEM) to characterize the biomaterial surface and to analyze the effects of BGMS10 addition on the alginate structure. eSEM imaging was performed on scaffold cross sections (Figure 2) to evaluate possible differences in structure between Ctrl-sc (A), BG6-sc (B), and BG12-sc (C) samples. At the microscopic level, the scaffold obtained after adding the bioactive glass (BG) maintained a rough surface due to the mineral component, similar to the Ctrl-sc. The figures (Figure 2) demonstrate that Alg/HAp-BGMS10 scaffolds display smooth areas through the interconnected cavities due to the presence of BG.

**Figure 3:**
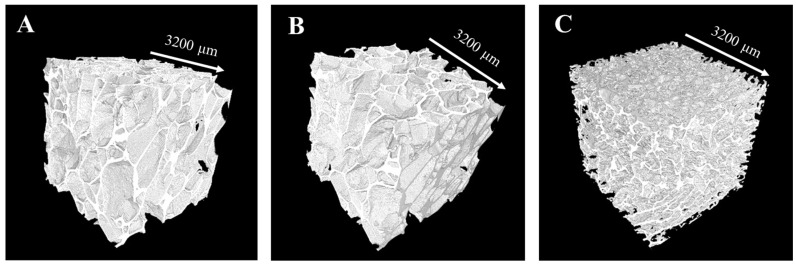
Three-dimensional reconstructions of Ctrl-sc (**A**), BG6-sc (**B**), and BG12-sc (**C**) determined with microcomputed tomography. Differences between in the porosity and pore distribution of BG12-sc with respect to Ctrl-sc and BG6-sc can be appreciated.

After assessing the microstructure of Ctrl-sc and BG-sc scaffolds through qualitative eSEM analysis, the three-dimensional structures were further examined to obtain more details about the morphological properties. Microcomputed tomography (μ-CT) of BG-sc prepared with the two different concentrations of BGMS10 was performed, and the results were compared to those obtained investigating Ctrl-sc. Referring to Table 2, the incorporation of BG into an alginate matrix decreased the microporosity of the alginate scaffold. Three-dimensional rendering of the samples (Figure 3) confirmed the similarity between the microstructures of Ctrl-sc and BG6-sc; therefore, this concentration was selected for further mechanical and biological characterizations of the composite scaffolds. In fact, the total porosity of the BG12-sc structure (≈70%) is lower than the porosity suggested for guided bone regeneration and may not be able to support optimal cell colonization.

**Figure 4:**
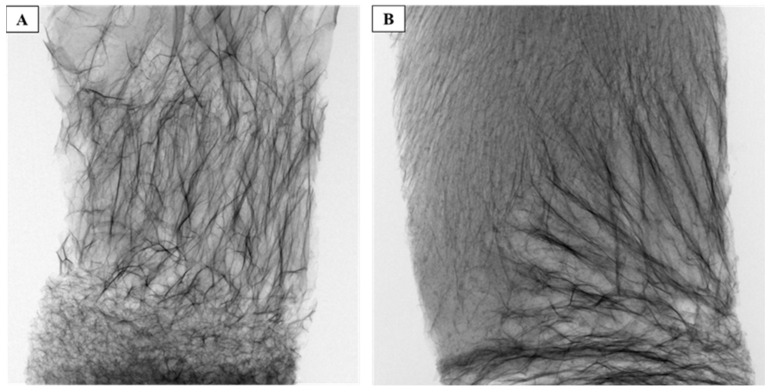
Projection of 45S5^®^6-sc (**A**) and NaCl-modified BG6d-sc (**B**) determined with μ-CT analysis. The images show the effect of the addition of NaCl to BG6-sc, which results in an alteration in scaffold porosity, similar to 45S5^®^6-sc.

As shown in Figure 4A, the 3D reconstruction of 45S5^®^6-sc shows that adding the commercial bioglass powder interferes with the scaffold formation, leading to an irregular and non-porous structure. The compositions of the different BGs mentioned in the introduction show a significant difference in sodium ion concentration between BGSM10 and 45S5^®^ powders, which could be responsible for the observed defects in the structure of the 45S5^®^6-sc. To investigate the effect of the sodium concentration, a composite BGSM10 scaffold with added NaCl was produced. The μ-CT analysis of this modified scaffold is reported in Figure 4B and confirms that sodium ions adversely affect the formation of the scaffold structure.

The 3D reconstructions of different portions of 45S5^®^6-sc and NaCl-added BG6d-sc are reported in Figure S1A and Figure S1B, respectively, showing the heterogeneity of the structures and porosity throughout the scaffolds.

**Figure 5:**
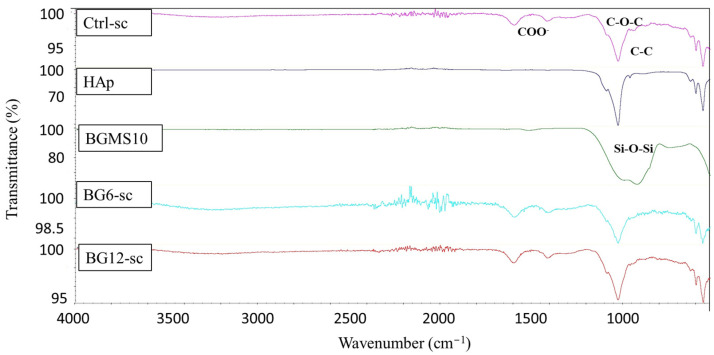
ATR-FTIR spectra of BG-sc compared with Ctrl-sc, pure BGMS10, and pure HAp powder; the spectra do not show any chemical alteration in scaffold composition.

The infrared spectra obtained from ATR-FTIR analysis of BGMS10 and HAp powder, as well as Ctrl-sc, BG6-sc, and BG12-sc, are presented in Figure 5. Pure alginate displays characteristic bands around 1591 cm^−1^ and 1418 cm^−1^ corresponding to COO^−^ groups of alginate, along with adsorption bands at 1079 cm^−1^ and 1026 cm^−1^, which correspond to C-O-C and C-C stretching bonds, respectively. The infrared spectra of BGMS10 show a vibration band at 1000 cm^−1^, representing a Si-O-Si asymmetric stretching bond. Comparing the spectra of BG6-sc and BG12-sc, with those of Ctrl-sc and pure BG powders suggests that the addition of BGMS10 does not affect characteristic bands of Ctrl-sc. To complete the study, ATR-FTIR spectra of 45S5^®^ powder and 45S5^®^6-sc are reported in Figure S2.

**Figure 6:**
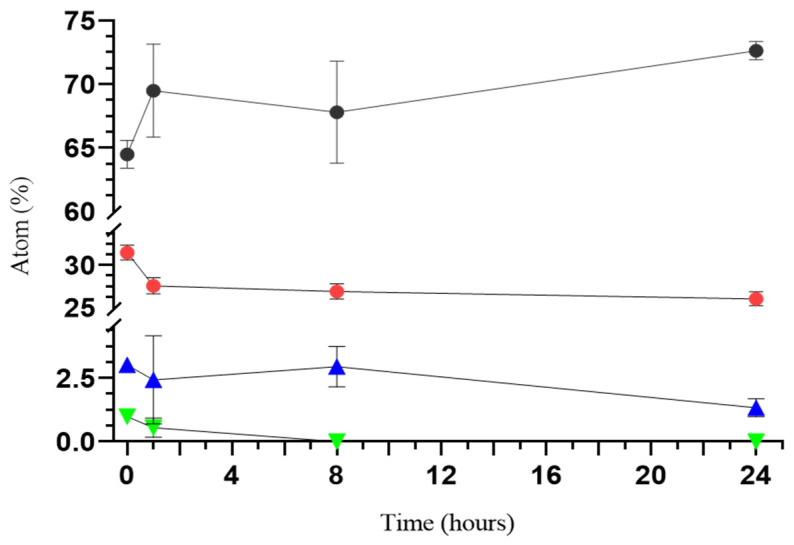
Concentrations (expressed as atomic percentages) of calcium (●), phosphorus (●), silicon (▲), and magnesium (▼) in BG6-sc after immersion in PBS for up to 24 h analyzed by microanalysis; slight decreases and releases of P, Mg, and Si are observable.

The variation of the concentrations of inorganic elements in BG6-sc was monitored over time by EDS analysis, immersing the scaffolds in PBS, as displayed in the graph in Figure 6. After 24 h, the phosphorus concentration decreased in the scaffold. Silicon levels remained relatively constant, with a slight decrease after 8 and 24 h, possibly due to an apatite formation process that consumes silicon ions. The concentration of magnesium, which is relatively low in the native BGMS10, significantly decreases in composite scaffolds over time.

### 3.2. Mechanical Characterization

**Figure 7:**
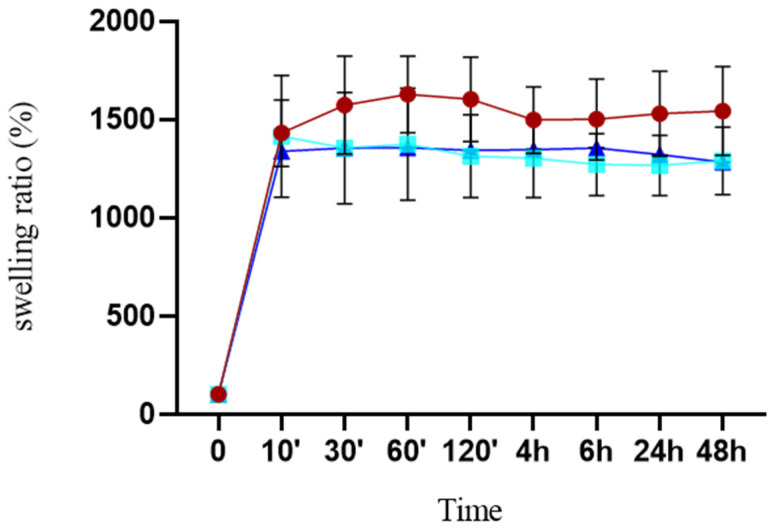
Swelling ratios of Ctrl-sc (●), BG6-sc (■), and BG12-sc (▲) after incubation in PBS at 37 °C. Error bars indicate the standard deviation calculated on 6 scaffolds (N = 6). There are no statistically significant differences in the behaviors of the scaffolds.

In order to be suitable for bone tissue engineering, it is essential for scaffolds to possess appropriate swelling properties. Composite scaffolds were immersed in PBS, and their weight changes were measured over time to determine their swelling behavior. The BG-loaded scaffold swelled quickly within 10 min and reached a constant swelling ratio. The graph in Figure 7 does not show any differences in the behaviors of BG6-sc and BG12-sc. The swelling ratio of Ctrl-sc was higher than that of the BG scaffolds and did not increase further after one hour. Adding BG decreased the water absorption ratio, which could be attributed to reductions in porosity and pore size.

**Figure 8:**
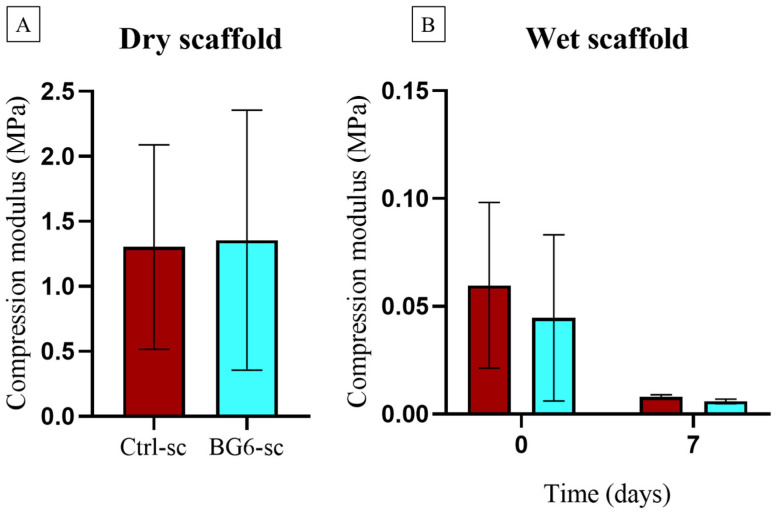
Elastic modulus evaluated on dry (**A**) and wet (**B**) Ctrl-sc (red) and BG6-sc (blue) at T0 and after 1 week of immersion in PBS. Statistical analysis was performed using the Mann-Whitney test for comparison of two groups, applying Bonferroni’s correction. Despite the differences between day 0 and day 7, due to the high variability of the measures, there were no statistical differences between the groups (N = 3).

Uniaxial compression tests performed on dry and wet scaffolds were used to test their mechanical behavior. Evaluations of elastic modulus (E) and ultimate compressive strength (σ_ucs_) show that Ctrl-sc and BG6-sc exhibit similar properties in terms of elastic modulus and ultimate compressive strength in the dry state. This suggests that adding 0.3% BGMS10 does not have an effect on the mechanical properties of the alginate/HAp scaffolds. As reported in Table 3, wet scaffolds exhibited lower mechanical resistance than the dry ones. As depicted in Figure 8, soaking in PBS causes a significant decrease in compressive modulus and ultimate compressive strength over a period of one week, resulting in weak mechanical resistance.

### 3.3. Biocompatibility and Cell Morphology on Scaffolds

To assess if composite scaffolds are able to support cell adhesion and proliferation and to test the biocompatibility of BGMS10, MG-63 cells were seeded on the scaffold. Their adhesion and growth were measured using Alamar Blue and MTT assays.

**Figure 9:**
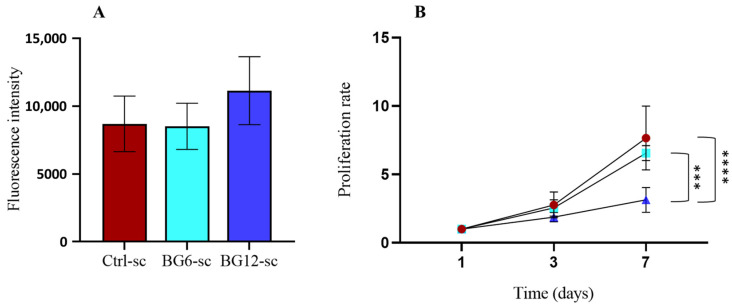
Biocompatibility of BGMS10 scaffolds in terms of MG-63 adhesion and proliferation. Statistical analysis was performed with ANOVA test, applying Bonferroni’s correction. Statistically significant differences are indicated with asterisks; *** = *p* < 0.001, **** = *p* < 0.0001. (**A**) Fluorescence intensity of Alamar Blue assay measured to evaluate MG-63 adhesion on scaffolds after 24 h; no statistically significant differences were observed. (**B**) Cell proliferation in Ctrl-sc (●), BG6-sc (■), and BG12-sc (▲); a clear trend of proliferation can be observed, with lower values for BG12-sc. The differences between the time points for the same sample are all statistically significant and are not reported for sake of clarity (N = 3).

Cell adhesion was evaluated 24 h after seeding, and no remarkable differences were detected (Figure 9A). Cells grown in a multiwell plate were used as controls for both types of scaffolds, and the fluorescence was normalized on day 1 to calculate the proliferation rate over time. The proliferation rates (Figure 9B) on BG6-sc and Ctrl-sc were comparable, although the microporosity of BG6-sc was slightly lower, as shown in the structural characterization. However, cell viability on BG12-sc was significantly reduced between day 3 and day 7 compared to BG6-sc and Ctrl-sc. This result could be attributed to the reduced porosity, which does not allow for cell proliferation within the scaffold, rather than lower material compatibility. Nevertheless, none of the scaffolds exhibited any cytotoxic effect towards the MG-63 cell line.

**Figure 10:**
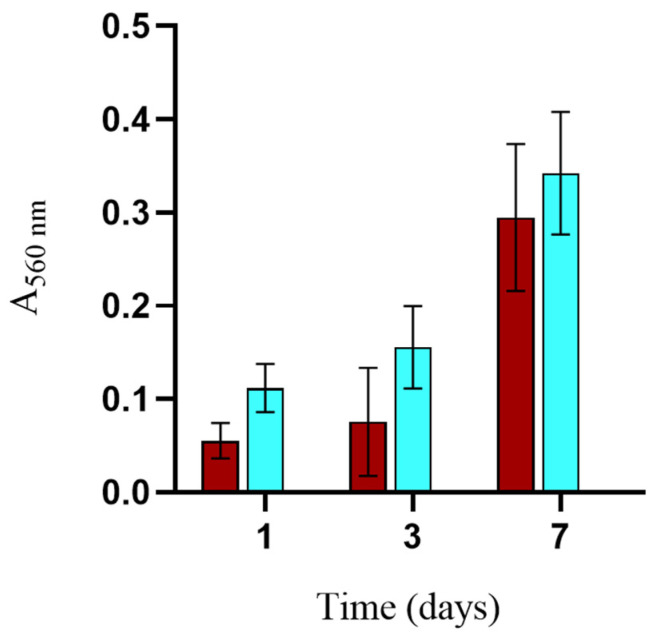
MG63 viability on Ctrl-sc (blue) and BG6-sc (red) scaffolds evaluated by MTT assay. Error bars represent the standard deviation of the average of 4 scaffolds tested at each time point. Statistical analysis was performed with ANOVA test, applying Bonferroni’s correction. A proliferation trend is appreciable over time; the differences between the time points for the same sample are all statistically significant and are not reported for sake of clarity. There were no statistical differences between the groups (N = 3).

To further investigate potential differences in cell proliferation within BG-loaded scaffolds, an MTT test was used to investigate cell viability. Considering its structural properties and the results of cell proliferation, BG6-sc was chosen to further investigate biocompatibility. The proliferation rate evaluated using the MTT test, as shown in Figure 10, demonstrates the positive effect of BG-sc on cell growth and scaffold colonization, with a trend comparable to that of Ctrl-sc.

**Figure 11:**
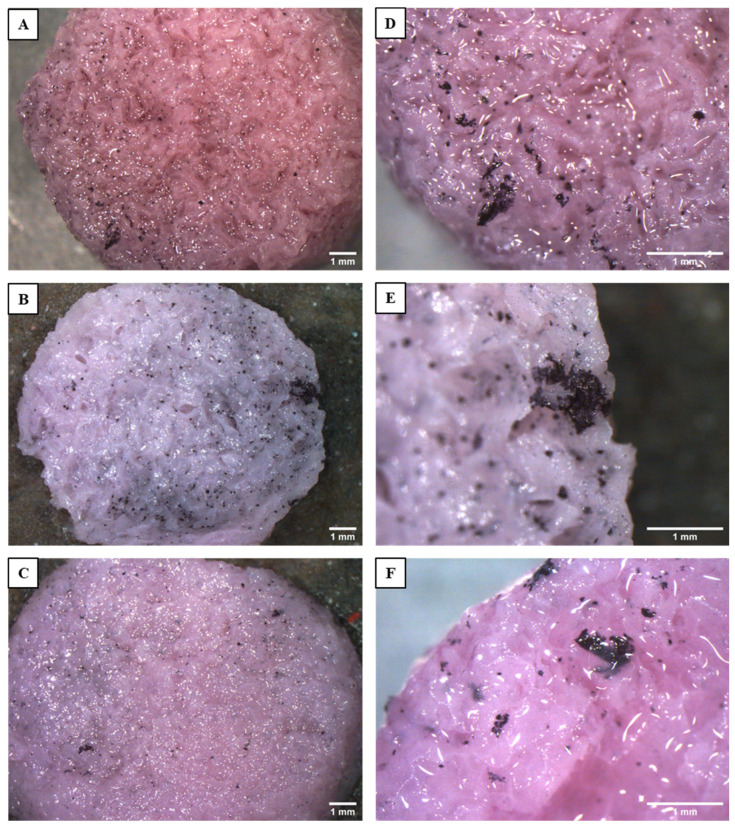
Top views of Ctrl-sc (**A**,**D**), BG6-sc (**B**,**E**), and BG12-sc (**C**,**F**) captured by stereoscope imaging at two different magnifications. The black spots correspond to cells and cell clusters, which metabolized the MTT dye. The higher level of cell colonization on BG6-sc is clearly visible.

MTT can also be used for qualitative assessment; indeed, viable cell clusters can be detected thanks to the reduction of soluble tetrazolium salt into insoluble purple formazan crystals. Thus, viable cell clusters were visualized using a stereoscope. On the last day of the viability test, stereoscope analysis confirmed the findings of the quantitative assays. Figure 11 shows top views of the scaffolds. MG-63 cells form a few isolated clusters on the surface of BG12-sc, probably due to the reduced porosity of the scaffold, while colonization is similar and better on Ctrl-sc and BG6-sc. At higher magnifications, it can be observed that cell clusters appear more numerous and larger on BG6-sc; however, further experiments are needed to verify these findings by enlarging sample size. Figure S3 shows that MG-63 cells are also able to colonize the inner portion of the scaffolds, confirming that the porosity and pore distribution of the scaffolds are suitable for bone tissue engineering applications.

**Figure 12:**
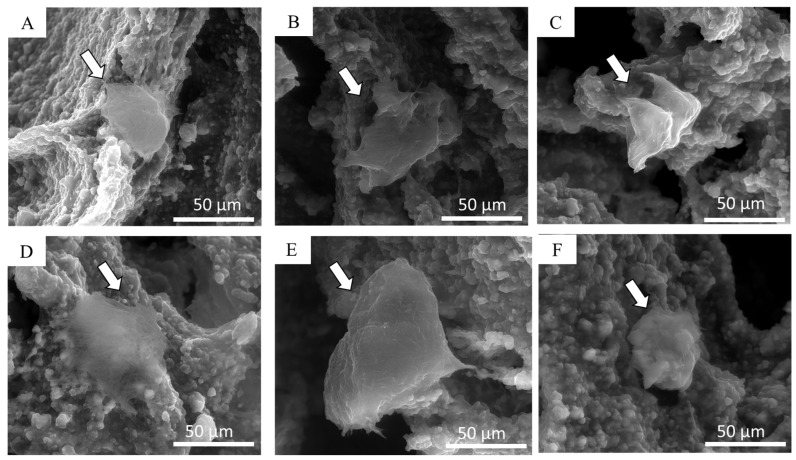
eSEM micrographs of MG-63 cells (indicated by arrows) seeded on Ctrl-sc (**A**), BG6-sc (**B**), and BG12-sc (**C**) on day 1. Cells spreading on Ctrl-sc (**D**), BG6-sc (**E**), and BG12-sc (**F**) on day 8. Differences in cell morphology between BG12-sc, BG6-sc, and Ctrl-sc can be appreciated.

Using environmental SEM imaging, it was possible to appreciate the morphology of the cultured cells after 24 h and one week of culture. eSEM micrographs (Figure 12A–C) clearly show adhered cells 1 day after seeding, which can be distinguished due to their smoother surfaces compared to the rough surface of the porous scaffold. MG-63 cells exhibited a spherical shape on all samples. After one week, cells were observed spreading across the scaffold trabeculae of Ctrl-sc, BG6-sc, and BG12-sc, proving the biocompatibility of BGMS10 (Figure 12D–F). In particular, MG-63 seems to adhere more favorably on the composite scaffold containing 0.3% *w*/*v* of BGMS10 than on scaffolds containing 0.6% BGMS10, where cells show a more rounded shape, confirming that BG12-sc may not be the most suitable scaffold for cell proliferation and bone tissue regeneration purposes.

### 3.4. Antibacterial Activity

A preliminary test of BGs ability to inhibit bacterial growth was performed using *S. aureus*. Liquid extracts of both BGMS10 and of 45S5^®^ powders were prepared and added to the bacteria; bacterial growth was followed by monitoring absorbance at 600 nm every 30 min. The results (Figure S4) reveal an optimal inhibitory effect of both BGs compared to the control strain.

**Figure 13:**
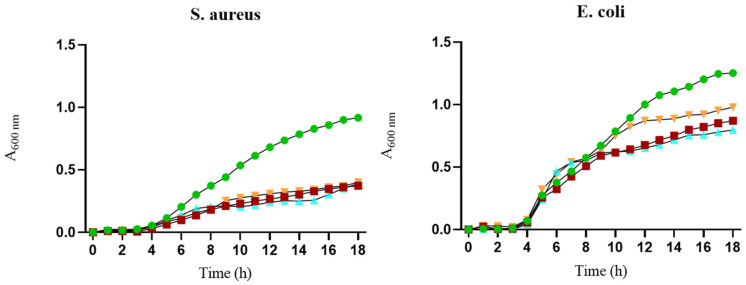
Bacterial growth expressed as absorbance at 600 nm for *E. coli* and *S. aureus* following exposure to extract solutions of Ctrl-sc (■), BG6-sc (▲), and 45S5^®^6-sc (▼) samples. Bacterial strains (●) indicate growth-positive controls (without scaffold extracts). Similar behavior can be observed between the tested samples, which differ from the control samples.

The potential antibacterial effects of the BGMS10 scaffold were tested against Gram-negative (*E. coli*) and Gram-positive (*S. aureus*) bacteria. Bacterial broth was conditioned with a scaffold to extract its elements and used for bacterial growth. The ability of the extracts to inhibit bacterial proliferation was measured by tracking bacterial growth kinetics. Figure 13 shows slightly higher bacterial growth inhibition over time compared to 45S5^®^6-sc for both *S. aureus* and *E. coli* when BG6-sc was used. Both the Ctrl alginate and 45S5^®^6-sc scaffolds exhibited slight antibacterial efficacy against both bacterial strains. Nevertheless, all tested scaffolds expressed an antimicrobial effect against *S. aureus* and *E. coli* when compared to the proliferation control (bacteria grown without any treatment).

## 4. Discussion

Alginate–hydroxyapatite three-dimensional porous scaffolds are a promising choice for bone tissue engineering applications. Alginate allows for the preparation of scaffolds that exhibit good biocompatibility and can be modified to regulate their morphological and biological properties, and enhance their bioactivity, without the drawbacks related to autografts such as morbidity and risks of the harvesting procedure [37]. For the purpose of bone regeneration, alginate (Alg) and hydroxyapatite (HAp) with augmented mineral components of the material were used for the preparation of porous structures in combination with an experimental bioactive glass (BG), BGMS10, synthesized as reported by Bellucci et al. [20]. Porous scaffolds were obtained by freeze drying Alg/HAp hydrogels obtained by a slow gelation method in which GDL was used to lower the pH of the solution, leading to the release of Ca^2+^ ions from HAp, the formation of alginate egg boxes, and the gelation of the alginate [9,38].

BGMS10 was developed with the following two main objectives: on one hand, to create a BG with a higher crystallization temperature compared to 45S5, which is the current commercial reference standard; and, on the other hand, to introduce magnesium and strontium into the glass composition, the beneficial effects of which have been widely reported, particularly in relation to bone tissue. Various combinations of these ions have been described in the literature, and the effect of each individual ion was investigated [21,22,23,39,40]. The result is a BG with a very high crystallization temperature (onset crystallization temperature: 880 °C; peak crystallization temperature: 932 °C [20]), which allows the glass to be sintered while preserving its amorphous nature, thus maintaining its bioactivity. For comparison, 45S5 crystallizes at temperatures around 600 °C when subjected to heat treatment to sinter the powders [41,42]. Beyond heat treatment, the excellent degrees of bioactivity, biocompatibility, and osteoconductivity of BGMS10 in granules have been tested in vitro and in vivo in an animal model (rabbits) [43].

In the present work, for the first time, powdered BGMS10 glass was used in conjunction with alginate to produce innovative scaffolds. The microstructures of the scaffolds were investigated, as porosity is a fundamental aspect in bone tissue engineering. To create a framework for bone growth, the acceptable range of porosity is 70–90%, and the minimum pore size is almost 100 µm (e.g., osteoblast cells have an average size of 20–25 µm) [44]. μ-CT analysis demonstrated that the addition of 0.3% *w*/*v* BGMS10 of the total gel volume, corresponding to 10% of the inorganic mineral part, does not alter the morphological features of the Alg/HAp scaffold developed by Turco and colleagues [10]. This protocol enables interconnected matrices to be obtained, which should facilitate cell colonization and proliferation, in addition to the exchange of nutrients and catabolites from the area. According to Fiume et al., incorporating BG into an alginate matrix slightly decreased the microporosity of Ctrl-sc. The modest difference in microstructure values may be attributed to the dimensions of ice crystals formed during hydrogel freezing, which may be influenced by the presence of BG and its interaction with water molecules [14].

Despite this, the porosity of the composite scaffolds implemented with BGMS10 is compatible with bone regeneration and should ensure cell spreading and proliferation [44]. Based on initial analysis, the parameters of the BG12-sc, including the total porosity of the structures (about 70%), were lower than the minimum value recommended for guided bone regeneration. This may not be ideal for cell ingrowth and migration. Therefore, the study proceeded focusing on the scaffold with the lowest BG concentration (6% of the dried scaffold). Environmental SEM imaging reveals that the pore walls of alginate/HAp-BGMS10 6% of the dried scaffold (BG6-sc) are substantially smoother than those of their Ctrl-sc counterparts. This phenomenon can be attributed to the dissolution of BGMS10, which prompts the precipitation of hydroxyapatite on the material surface [45].

When compared to BGMS10, 45S5 Bioglass ^®^ contains significantly more sodium. As a preliminary hypothesis, it can be stated that the increased release of sodium ions from 45S5^®^ in the acidic environment of alginate gelation [46] could hamper the binding of calcium ions with the carboxylate groups of the G blocks of alginate, thus impeding the formation of egg boxes and disrupting the gelation mechanism of alginate hydrogel [47]. This interference can result in an irregular and inconsistent structure across different planes, as observed when BG6-sc is prepared by adding 10% NaCl.

The ATR-FTIR spectra indicate that the BG-enriched scaffold retains the properties of the original alginate scaffold, as reported in the literature. Comparison of the characteristic bands of the composite with those of the alginate material and BGMS10 powders containing Sr^2+^ and Mg^2+^ reveals a vibration located at 1000 cm^−1^, which is assigned to the Si-O-Si asymmetric stretching bond. This suggests that adding ceramic powder does not interfere with the physicochemical interaction of alginate and hydroxyapatite porous scaffolds.

The incorporation of BGs has been found to boost alkaline phosphatase (ALP) activity, cell adhesion, and proliferation through the release of Si, Ca, Na, and P ions, which are responsible of the activation and upregulation of gene expression in cells [48], thereby enhancing the biological performance of our composites. It is crucial to thoroughly examine ion release and its effects on the investigated biomaterials. Released silicon levels exhibited minimal variation over time, with a slight increase after 1 h and decreases after 8 and 24 h. This could be attributed to the apatite formation process, which may or may not consume silicon ions [14]. The concentration of magnesium, which was initially low in the native BGMS10, decreased further over time in composite scaffolds. However, even though the concentration is low, the presence of magnesium can benefit the composites’ biological and mechanical performance. Magnesium ions are known to have an effect on cell proliferation and differentiation and play an essential role in the metabolism of bone tissue [27]. The strontium ions contained in BGMS10 promote osteogenic and osteoblast differentiation [26]; however, it is necessary to establish if strontium ions released from the composite scaffolds are sufficient to improve cell viability and, potentially, combat bacterial strains effectively.

The mechanical strength of bone substitutes is crucial for implant success, as their 3D structures must be maintained both during surgery and after transplantation [49]. Dry BG6-sc exhibited similar mechanical behavior to that of Ctrl-sc, as reported by Turco and colleagues [9,10]. However, its mechanical performance significantly decreased after wetting, highlighting the need to optimize the porous structure to withstand natural stresses on bone tissue [50]. Indeed, biomaterials used in critical and load-bearing bone defects require adequate mechanical strength to mimic native tissue properties and facilitate the formation of new bone without compromising mechanical and functional performance. Achieving this goal with polymeric materials poses a significant challenge.

The mechanical characteristics of the scaffolds discussed here do not match those of native bone tissue [51]; indeed, a biodegradable porous composite studied for cancellous bone replacement exhibited a compressive strength in the range of ∼0.015–∼1 MPa [52]. Thus, the polymer-based porous scaffolds presented in this work might be suitable for repairing non-critical bone defects [53]. Meanwhile, porous scaffolds should be able to uptake water and nutrients for cell growth while exhibiting controlled swelling behavior that does not compromise their mechanical properties. Composite scaffolds quickly swelled in PBS and reached a constant swelling rate. This rapid uptake may positively impact cell adhesion and growth [23,54,55]. The presence of BG reduces the surface area of the polymer, thereby hindering its ability to bind water molecules. Gentile’s study conducted in 2017 confirmed that the swelling degree decreases when the BG content increases [56]. Furthermore, the decrease in water uptake could be attributed to reductions in both porosity and pore size, as suggested by Fiume et al. [14].

After characterizing their physicochemical and mechanical properties, the preliminary biocompatibility of the composite porous structures was assessed using the MG-63 cell line as an ideal cellular model. These cells are a well-established human osteoblast model commonly utilized for in vitro analysis of bone implants. Notably, they maintain stable phenotypic characteristics even after extended cell culture passages [57]. All the scaffolds proved to be biocompatible toward the MG-63 cell line, with a similar trend observed across all scaffolds. Despite the slight decrease in porosity with the addition of BGMS10 content, a proliferation rate comparable to that of Ctrl-sc was observed. This could be due to the dissolution of ions that stimulate cell attachment and proliferation. Specifically, bioactive Mg^2+^ and Sr^2+^ ions might enhance and ameliorate the bioactivity of composites compared to alginate scaffolds [23], as previously mentioned.

Cell adhesion and subsequent proliferation were evaluated on composite scaffolds using two assays commonly employed in tissue engineering studies, namely Alamar Blue and MTT assays. While Alamar Blue is compatible and non-toxic, it has limitations in 3D cell culturing, as it can underestimate the number of cells in the biomaterial. This is due to the possibility of high cell concentrations depleting the resazurin pool, which can affect the accuracy of the correlation between resazurin reduction and viable cell number [58]. Therefore, MG-63 proliferation rates were confirmed using an MTT assay, which also enabled imaging of scaffold cell colonization thanks to the reduction of soluble tetrazolium to insoluble purple formazan crystals.

Following a preliminary incubation stage, the MG-63 cells initially adhered in a spherical form before eventually extending across the scaffold surface. Despite the reduced porosity, the release of therapeutic ions from BG encouraged osteoblast cell adhesion and growth. By the end of the first week of culture, the cells had successfully adhered, dispersed, and proliferated across the trabecular surface. Future studies should investigate the ability of magnesium and strontium to stimulate osteogenic differentiation in a cell line suitable for bone regeneration, as well as their roles in bone metabolism, by evaluating specific biomarkers of bone differentiation and extracellular matrix deposition. Moreover, further investigations are needed to better evaluate and measure cell colonization and spreading within the scaffolds, possibly by seeding the scaffolds under dynamic conditions, e.g., by using perfusion bioreactors to better mimic the physiological environment [59].

Given the urgent need for devices that can lower the risk of infection following implantation procedures, the development of new bone grafts enriched with antibacterial properties was pursued. The antibacterial efficacy of composite scaffold matrices was tested against *E. coli* and *S. aureus* reference strains, which are clinically relevant pathogens. The inhibitory activity of BGMS10 and 45S5^®^ powders was first assessed by exposing them to *S. aureus*. The study showed that both BGs were highly effective in inhibiting the growth of this bacterial strain. These findings further support the already established antibacterial properties of commercial 45S5^®^ [6]. Bactericidal activity was tested using the BG-loaded scaffolds against both strains, and it was observed that all scaffolds exhibited similar antibacterial performance. Based on these findings and supported by several studies that have reported on the antibacterial properties of biomaterials and compounds including HAp [60,61], it can be hypothesized that the mineral component of the scaffolds may be responsible for the observed slowdown in bacterial growth. The concentration of calcium phosphate components could potentially explain the observed antibacterial behavior, as reported by Souad and Baghdadi in 2020 [62]. Additionally, the ions released from the surface disrupt the bacterial membrane potential, leading to increased osmotic pressure, subsequent disruption of the bacterial outer membrane, and cell death [63]. Instead, there is a difference in the way that Gram-negative bacteria respond to scaffolds, as indicated by the minimal slowdown observed for scaffolds compared to the positive growth control. The Gram-negative cell wall contains a higher proportion of phospholipids, lipopolysaccharides, and proteins with respect to the Gram-positive cell wall, which has peptidoglycan as its main component [64]. This difference in composition may have an effect on the antibacterial effects of the ions [6]. However, further experiments are required to confirm this hypothesis. Furthermore, strontium exhibits weak antibacterial activity and cariostatic properties [33]. The release of Sr from BGMS10 may affect potential bacterial growth. Further investigations are needed to clarify the unexpected and interesting antimicrobial activity of Ctrl-sc samples towards the tested bacterial strains. Further studies should be performed to calculate the MIC (Minimum Inhibitory Concentration) and MBC (Minimum Bactericidal Concentration) to understand the link between bioglass concentration and antibacterial effects.

## 5. Conclusions

The composition of bioactive glasses (BGs) can be customized by adding bioactive ions to trigger biological responses in the patient or introduce new functionalities. In this context, we demonstrated that it is possible to create porous and biocompatible composite scaffolds combining alginate/hydroxyapatite and an experimental BG named BGMS10, containing strontium and magnesium. We found that using the gold-standard 45S5 Bioglass ^®^ in the same manufacturing process was unsuccessful in obtaining a porous structure suitable for biological studies. The lower tested concentration of BGMS10 did not significantly alter the scaffold structure, slightly reducing its porosity and mechanical properties. Despite the slightly smaller surface area available for cell colonization, the composite scaffold was shown to be able to support MG-63 cell adhesion, colonization, and proliferation. Antibacterial activity assays indicated that the scaffold’s ion composition interferes with the proliferation of both *S. aureus* and *E. coli* strains.

The data revealed that an alginate/hydroxyapatite-BGMS10 porous scaffold is suitable for potential applications in bone tissue engineering. The limitations of this study are mainly related to the necessity of further investigations of the behavior of an osteoblast cell line within the scaffolds in terms of both cell spreading and scaffold colonization and of cell differentiation and extracellular bone matrix synthesis. We are currently conducting cellular differentiation studies to further investigate the biological properties of composite scaffolds. Moreover, antimicrobial properties will be deeply investigated by better exploring the fine tuning of the bioactive glass concentration and composition to develop a material that can slow down or prevent bacterial infections.

## Figures and Tables

**Table 1:** Compositions (% *w*/*w*) of the dried scaffolds.

	Alginate	Hydroxyapatite	45S5^®^	BGMS10	NaCl
Ctrl-sc	40	60	-	-	-
BG6-sc	40	54		6	
BG12-sc	40	48		12	
45S5^®^6-sc	40	54		6	
BG6d-sc	36.3	49.1	-	5.5	9.1

**Table 2:** Quantitative characterization of the microstructures of the composite scaffolds.

	Ctrl-sc	BG6-sc	BG12-sc
Porosity	84.6 ± 0.3	80.2 ± 1.1	70.2 ± 0.6
Mean Tb.Th (µm)	43.9 ± 1.2	53.7 ± 10.1	61.5 ± 11.1
Mean Tb.Sp (µm)	328 ± 19.9	335.2 ± 29.1	217.7 ± 93.3
Conn.D (µm^−3^)	5.90 × 10^−8^ ± 0.54 × 10^−8^	3.72 × 10^−8^ ± 0.70 × 10^−8^	3.50 × 10^−8^ ± 0.40 × 10^−8^
DA	0.34 ± 0.1	0.41 ± 0.09	0.45 ± 0.1

Tb.Th: trabecular thickness; Tb.Sp: trabecular spacing; Conn.D: connectivity density; DA: degree of anisotropy. Data are reported as averages ± s.d. (N = 4). The linear resolution is 8 μm.

**Table 3:** Compression tests on dry and wet (at T0 and after 1 week of immersion) Ctrl-sc and BG6-sc. Elastic modulus (E) and ultimate compressive strength are reported as averages ± s.d. (N = 3).

	Ctrl-sc			BG6-sc		
	Dry	Wet (T0)	1 Week	Dry	Wet (T0)	1 Week
E (MPa)	1.30 ± 0.48	0.05 ± 0.03	0.008 ± 0.001	1.35 ± 1.02	0.04 ± 0.03	0.005 ± 0.001
σ_ucs_ (MPa)	0.20 ± 0.02	0.008 ± 0.001	0.003 ± 0.001	0.19 ± 0.05	0.011 ± 0.006	0.003 ± 0.001

## Data Availability

The raw data supporting the conclusions of this article will be made available by the authors upon request.

## Supplementary Materials

The following supporting information can be downloaded at: https://www.mdpi.com/article/10.3390/jfb15070183/s1, Figure S1: Segmentation of 3D reconstruction by µ-CT analysis; Figure S2: ATR-FTIR spectra of 45S5^®^6-sc compared with Ctrl-sc, pure 45S5^®^, and pure HAp powder; Figure S3: Growth inhibition of *S. aureus* following exposure to extract solutions of BGMS10 and 45S5^®^. Figure S4: Growth inhibition of *S. aureus* following exposure to extract solution of BGMS10 (▪, blue markers) and 45S5® (▲, orange markers) bioactive glasses, observed over time up to 20 h. *S. aureus* (●, green markers) indicates growth-positive control (without bioactive glass extracts).
