# Supplementary material for: Alginate-Sr/Mg Containing Bioactive Glass Scaffolds: The Characterization of a New 3D Composite for Bone Tissue Engineering
Source: J Funct Biomater. 2024 Jul 2;15(7):183. doi: 10.3390/jfb15070183 (PMC11278315; doi:10.3390/jfb15070183)
Supplement: Supplementary file 1 [file jfb-15-00183-s001.zip › jfb-3078872-supplementary.pdf]

Journal of
Functional
Biomaterials

MDPI

Alginate-Sr/Mg Containing Bioactive Glass Scaffolds: The Characterization of a New 3D Composite for Bone Tissue Engineering

SUPPLEMENTARY MATERIALS

1. Structural characterization of 45S5®6-sc and BG6d-sc

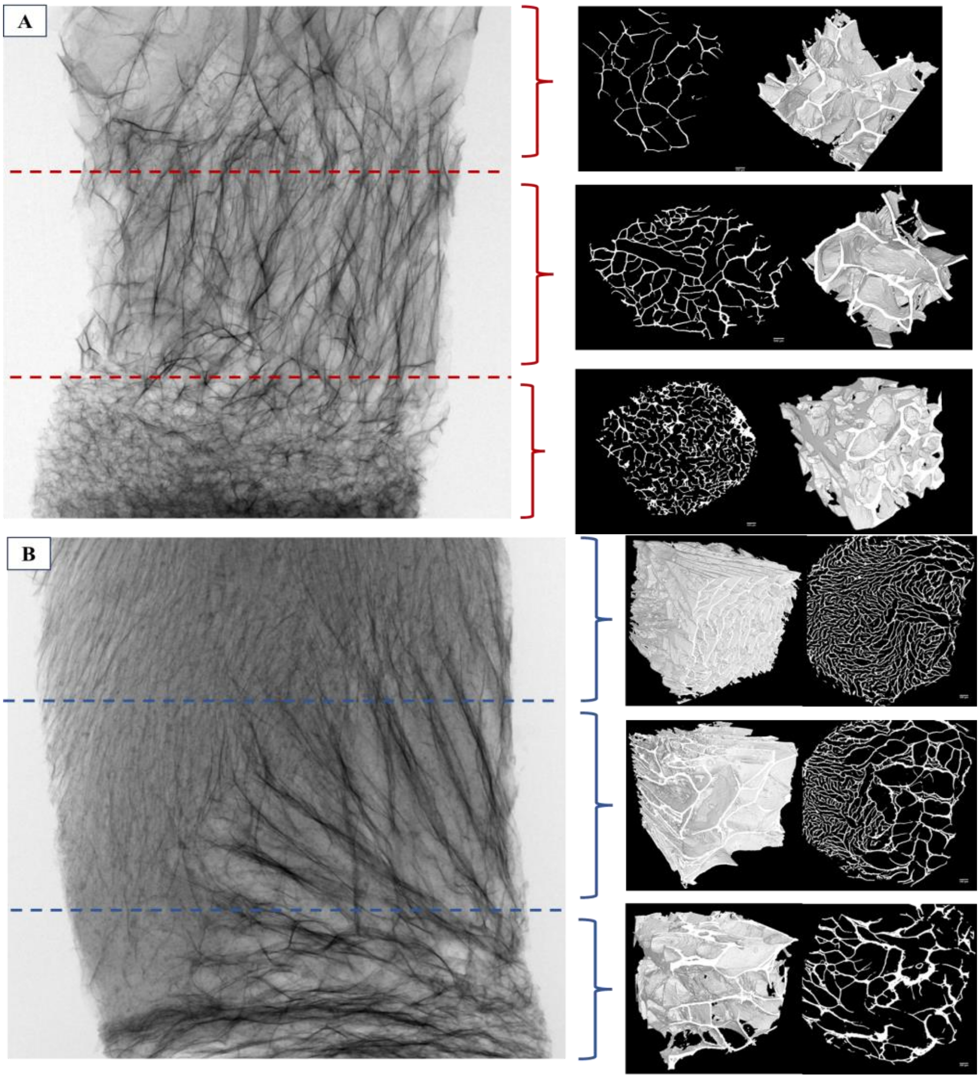

Figure S1. Segmentation of 3D reconstruction by μ-CT analysis at different heights (bottom, central and top part) of 45S5®6-sc (**A**) and BG6d-sc (**B**).

2. ATR-FTIR of 45S5® powder and 45S5®6-sc

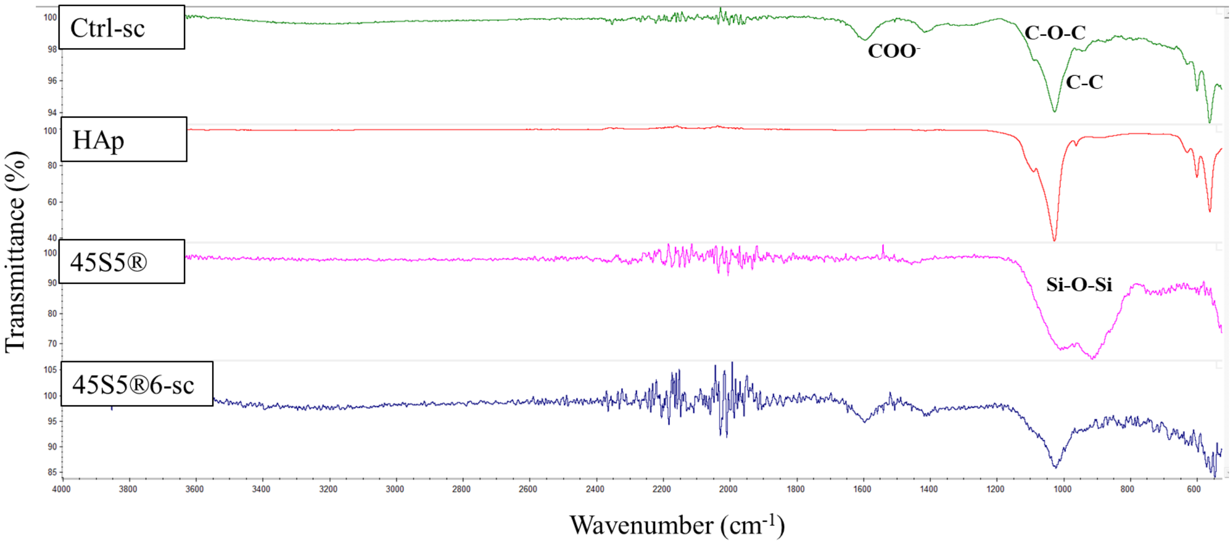

Figure S2. ATR-FTIR spectra of 45S5®6-sc compared with Ctrl-sc, pure 45S5®, and pure HAp powder.

3. Cell colonization of Ctrl-sc and BG6-sc

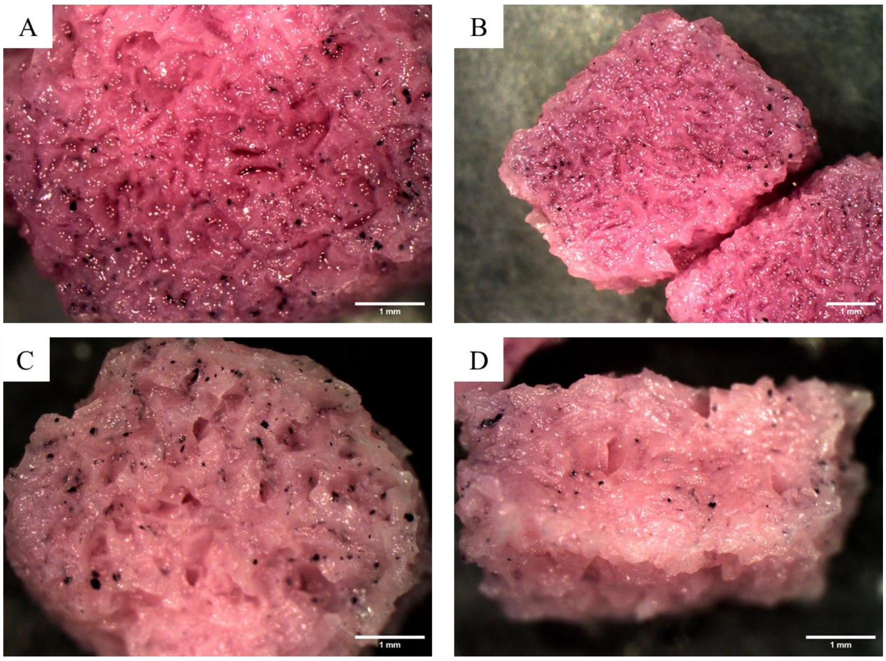

Figure S3. Top-view of Ctrl-sc (A) and BG6-sc (C) by stereoscope imaging. Internal view of Ctrl-sc (B) and BG6-sc (D).

4. Preliminary antibacterial assay

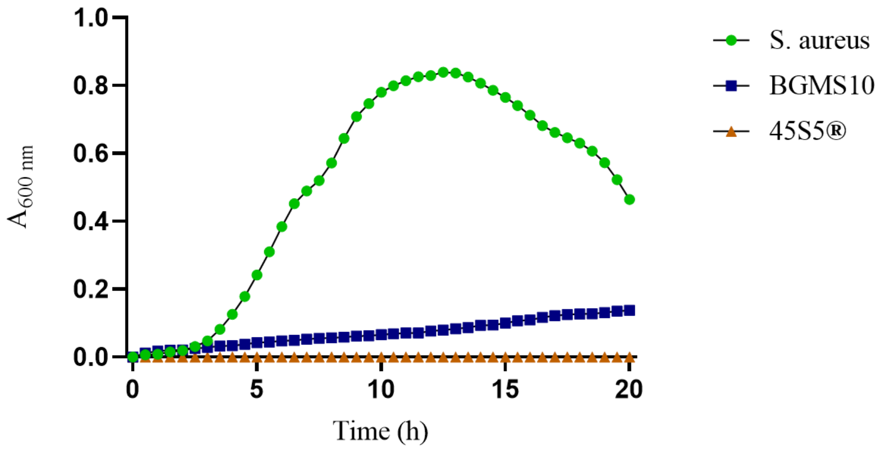

Figure S4. Growth inhibition of *S. aureus* following exposure to extract solution of BGMS10 (▪, blue markers) and 45S5® (▲, orange markers) bioactive glasses, observed over time up to 20 h. *S. aureus* (●, green markers) indicates growth-positive control (without bioactive glass extracts).
